# Therapeutic outcomes and prognostic factors in unresectable gallbladder cancer treated with gemcitabine plus cisplatin

**DOI:** 10.1186/s12885-018-5211-y

**Published:** 2019-01-05

**Authors:** Min su You, Ji Kon Ryu, Young Hoon Choi, Jin Ho Choi, Gunn Huh, Woo Hyun Paik, Sang Hyub Lee, Yong-Tae Kim

**Affiliations:** Department of Internal Medicine and Liver Research Institute, Seoul National University College of Medicine, Seoul National University Hospital, 101 Daehak-ro, Jongno-gu, Seoul, 110-744 South Korea

**Keywords:** Gallbladder neoplasms, Gemcitabine, Cisplatin, Prognosis, Treatment outcome

## Abstract

**Background:**

Gallbladder cancer (GBC) is likely to be diagnosed at progressive stages and shows a very poor prognosis. Combination therapy with gemcitabine and cisplatin (GEMCIS) has been widely used as first-line palliative chemotherapy for advanced GBC. This study was designed to investigate the efficacy of GEMCIS and identify prognostic factors in patients with unresectable GBC.

**Methods:**

Patients with GBC who were treated with GEMCIS from January 2008 to June 2017 in a single tertiary hospital were included. All cases of GBC were diagnosed by pathologic findings and extent of the tumour was assessed by imaging tests. Combination chemotherapy consisted of cisplatin 25 mg/m^2^ and gemcitabine 1000 mg/m^2^ intravenously on days 1 and 8 every 3 weeks. To determine factors affecting prognosis, Kaplan–Meier survival analysis, log-rank test and the Cox proportional hazard regression linear model were used. All variables with *P* < 0.1 in univariable analysis were included in the multivariable model.

**Results:**

A total of 173 patients received a median of 5.3 ± 4.4 cycles of chemotherapy over 3.8 ± 3.9 months. Most of the patients (94.8%) were stage IVB at the time of diagnosis and the most common site of metastasis was the liver (42.8%). Disease control rate was 59.5%: 2 (1.2%) patients with complete response, 26 (15.0%) patients with partial response and 75 (43.4%) patients with stable disease. Overall survival (OS) and progression-free survival were 8.1 (95% confidence interval [CI], 7.1–10.2) and 5.6 (95% CI 4.5–6.8) months, respectively. Multivariable regression model indicated that metastasis to liver (hazard ratio [HR] = 1.63, 95% CI 1.11–2.40; *P* = 0.013), neutrophil-to-lymphocyte ratio (NLR) ≥3 (HR 1.65, 95% CI 1.09–2.49; *P* = 0.017), CEA ≥ 5 ng/mL (HR 1.50, 95% CI 1.02–2.19; *P* = 0.038), and CA19–9 ≥ 500 U/mL (HR 1.59, 95% CI 1.01–2.50; *P* = 0.043) were significantly associated with OS.

**Conclusions:**

GEMCIS demonstrated a high disease control rate in patients with unresectable GBC. Factors independently related to OS were metastasis to liver, NLR ≥ 3, CEA ≥ 5 ng/mL and CA19–9 ≥ 500 U/mL.

## Background

Gallbladder cancer (GBC) accounts for 80 to 90% of carcinomas in the biliary system. Cancer-related mortality among patients with GBC is increasing and expected to continue to increase until 2030 worldwide [1]. Since there are no specific symptoms in the early stage, GBC is often not diagnosed until advanced stages. Surgery is the only curative treatment method; however, fewer than 10% of patients can undergo surgery, and 50% are found to have metastasis to lymph nodes at the time of diagnosis [2]. GBC has an abysmal prognosis and a median survival of 6 months if untreated [3].

Gemcitabine plus cisplatin (GEMCIS) is widely used as first-line chemotherapy for unresectable GBC based on a recent clinical trial showing favourable outcomes of the combination chemotherapy in patients with biliary tract cancer (BTC) [4]. Median overall survival (OS) and progression-free survival (PFS) were 11.7 and 8.0 months, respectively, in patients with BTC treated with GEMCIS [4]. However, in a more recent phase II clinical study that included only patients with unresectable GBC, median OS and PFS were 6.2 and 3.1 months, respectively [5]. Another study revealed that gallbladder cancer responds poorly to chemotherapy compared to other subtypes of biliary tract cancer [6]. Even though prognosis and response to chemotherapy in patients with GBC are different from those in patients with other subtypes of BTC, there is a lack of studies evaluating the efficacy of GEMCIS only in patients with GBC.

Several studies have identified various prognostic factors in patients with GBC, including jaundice, metastasis to lymph nodes, and metastasis to liver [7–10]. Tumour markers, particularly carcinoembryonic antigen (CEA) and carbohydrate antigen 19–9 (CA19–9), are also well-known prognostic factors in GBC [11–13]. In addition, it has been shown that several variables of systemic inflammation response such as neutrophil-to-lymphocyte ratio (NLR) and platelet-to-lymphocyte ratio (PLR) have prognostic value in BTC [14–16]. However, previous studies are based on retrospective data from patients with BTC or GBC who were treated surgically, and prognostic factors in patients with unresectable GBC treated with GEMCIS are largely unknown.

Although GEMCIS is widely used in patients with advanced GBC based on the clinical trial in 2010, there may be differences in treatment efficacy between GBC and other subtypes of BTC. Moreover, previous studies regarding treatment efficacy and prognostic factors have focused mainly on patients with BTC or GBC who were treated primarily by surgery. Therefore, this study aimed to evaluate the efficacy of GEMCIS and identify prognostic factors in only patients with unresectable GBC.

## Methods

### Study subjects

Patients with unresectable GBC who were treated with GEMCIS at Seoul National University Hospital between January 2008 and June 2017 were analysed. All patients were diagnosed by pathologic confirmation and medical records of the patients were retrospectively reviewed. Extent of the disease was evaluated by contrast-enhanced computed tomography and 18F-fluoro-2-deoxy-D-glucose positron emission tomography with computed tomography scan.

**Fig. 1:**
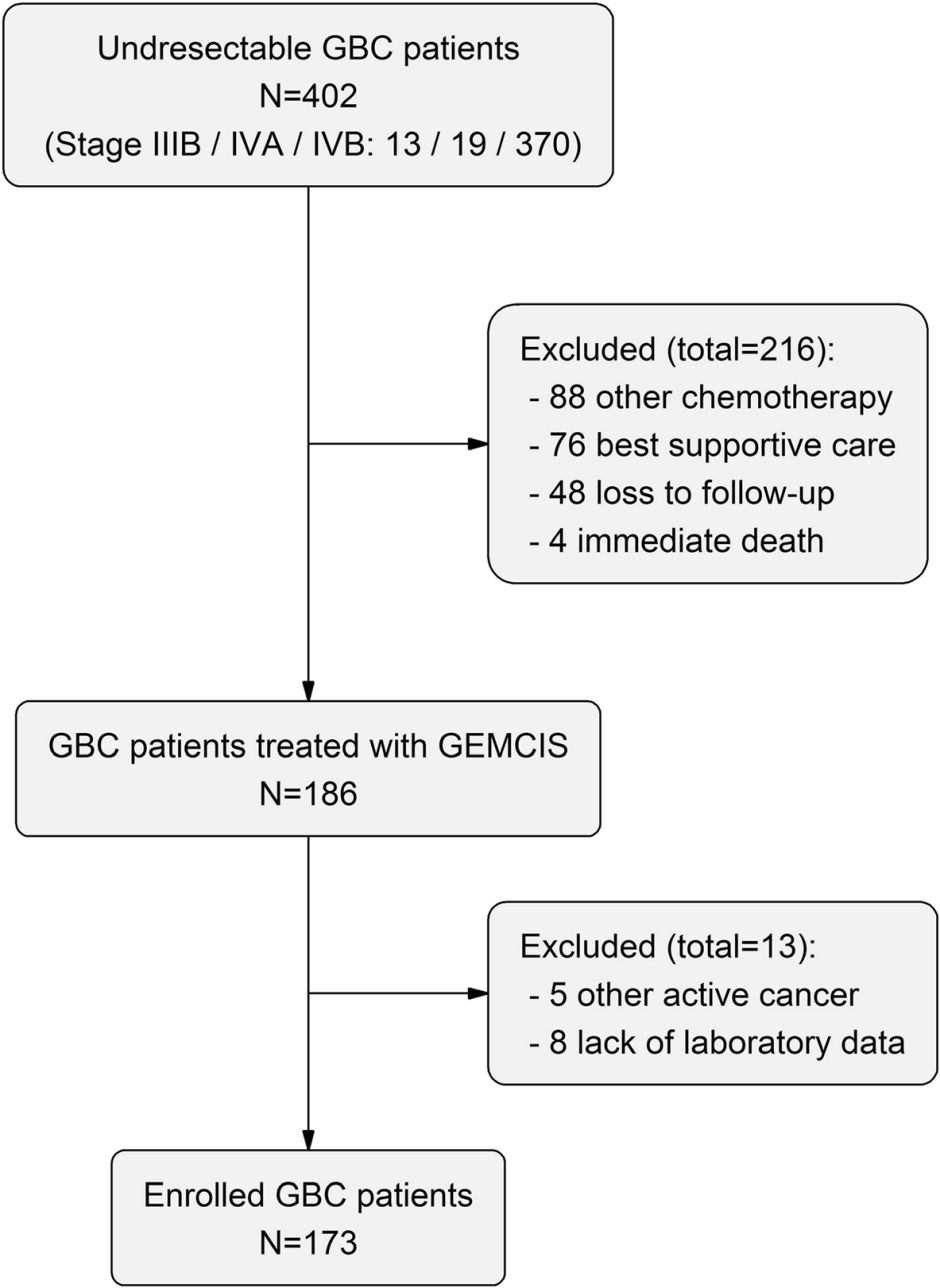
Flow chart of patient enrolment between January 2008 and June 2017. GBC, gallbladder cancer; GEMCIS, gemcitabine plus cisplatin

Locations of regional lymph nodes included hepatic hilus along the common bile duct, hepatic artery, portal vein, and cystic duct, whereas involvement of lymph nodes around the celiac and superior mesenteric artery was considered distant metastatic disease [17]. A total of 186 patients were reviewed. Five patients who lacked baseline laboratory findings and eight patients with history of active cancer in another organ within 5 years were excluded (Fig. 1). In total, 173 patients were enrolled and analysed. This study was approved by the Institutional Review Board of the Seoul National University Hospital, Seoul, Korea (1703–004-834).

### Treatment and data collection

Each cycle of combination chemotherapy consisted of cisplatin 25 mg/m^2^ and gemcitabine 1000 mg/m^2^ administered intravenously on days 1 and 8 every 3 weeks. The treatment was repeated until the occurrence of unacceptable toxicity, loss to follow-up, confirmation of disease progression, or death. Patients were followed up regularly and laboratory tests and imaging tests were periodically evaluated to assess therapeutic outcomes.

Demographic and clinical variables included age, gender, Eastern Cooperative Oncology Group (ECOG) performance status, body mass index, Charlson comorbidity index score, cancer stage, location of invasion and metastasis, previous therapeutic history, and total number of chemotherapy cycles. Laboratory variables included glomerular filtration rate, aspartate aminotransferase, alanine aminotransferase, total bilirubin, alkaline phosphatase, CEA, CA19–9, NLR, and PLR. NLR and PLR values were defined as the number of absolute neutrophils and platelets divided by the absolute lymphocyte count from samples of peripheral blood, respectively.

The primary endpoint was OS and the secondary endpoints were PFS and objective best overall tumour response. OS was defined as time from initiation of chemotherapy to the final date of follow-up or death from any cause. PFS was defined as time from initial treatment to the confirmation of disease progression or death. Mortality data were collected by the Ministry of the Interior and Safety. Best overall tumour response was assessed by contrast-enhanced CT scan based on the Response Evaluation Criteria in Solid Tumors (RECIST) 1.1 criteria [18]. Minimum duration for definition of stable disease was 1 month and tumour response was evaluated at intervals of two cycles.

### Statistical analysis

Continuous variables with a normal distribution were expressed as the mean and standard deviation, while those with a non-normal distribution were summarised as the median and interquartile range (IQR). Categorical variables were presented with percentage, and the Pearson χ^2^ test was applied to determine differences between them. OS and PFS were calculated and compared using the Kaplan–Meier method and the log-rank test. To determine factors affecting prognosis, hazard ratio (HR) and 95% confidence interval (CI) for OS and PFS were calculated using the Cox’s proportional hazard regression model. Best cut-off values of NLR, PLR, and tumour markers were obtained using the Contal and O’Quigley method [19]. Multivariable analysis was performed including variables with *P* < 0.1 in univariable analysis. Variables with *P* < 0.05 were considered to indicate statistical significance. All statistical analyses were performed using R ver. 3.3.3 (Institute for Statistics and Mathematics, Vienna, Austria; http://www.R-project.org).

## Results

### Clinical characteristics of patients

Baseline characteristics are summarised in Table 1. The most common site of adjacent cancer invasion was the liver (27.2%), followed by the extrahepatic bile duct (15.0%) and intestine (11.6%). Invasion of hepatic artery and main portal vein was identified in 15 (8.7%) and 11 (6.4%) patients, respectively. Twenty-seven (15.6%) patients were identified to have invasion to more than two organs. Most of the patients (94.8%) were in stage IVB. Among the total patients, liver metastasis was identified in 74 (42.8%) patients, followed by peritoneum in 46 (26.6%) and lung in 29 (16.8%) patients. There were two patients each with metastasis to abdominal wall and adrenal gland and one patient with metastasis to spleen. Metastasis to distant lymph node was found in 102 (59.0%) patients, pericaval area in 58 (33.6%), paraaortic area in 65 (37.6%), and the area above the diaphragm in 31 (17.9%) patients. Eighty-one (46.8%) patients had metastasis to multiple lymph nodes.

**Table 1:** Baseline characteristics of all patients

Variables	*N* = 173
Age (range)	63.8 (25.0–84.0)
Sex (female / male)	86 (49.7%) / 87 (50.3%)
ECOG (0 / 1 / 2)	36 (20.8%) / 126 (72.8%) / 11 (6.4%)
BMI	23.0 ± 2.8
Charlson comorbidity index	8.0 ± 1.4
Stage (IIIB / IVA / IVB)	1 (0.6%) / 8 (4.6%) / 164 (94.8%)
Invasion	
Liver	47 (27.2%)
Extrahepatic bile duct	26 (15.0%)
Intestine	20 (11.6%)
Peritoneum	2 (1.2%)
Hepatic artery	15 (8.7%)
Main portal vein	11 (6.4%)
Metastasis	
Liver	74 (42.8%)
Peritoneum	46 (26.6%)
Lung	29 (16.8%)
Bone or muscular system	14 (8.1%)
Distant lymph node	102 (59.0%)
Previous history	
Biliary drainage	49 (28.3%)
Curative surgery	33 (19.1%)
Palliative chemotherapy	12 (8.5%)
Baseline laboratory findings	
WBC (cells/μL)	6540.0 ± 5192.4
CRP (mg/dL)	3.5 ± 4.0
eGFR (mL/min/1.73m^2^)	92.8 ± 23.1
AST (IU/L)	40.3 ± 40.3
ALT (IU/L)	44.2 ± 50.8
ALP (IU/L)	183.7 ± 174.9
Bilirubin, total (mg/dL)	1.5 ± 2.4
CEA (ng/mL)	68.6 ± 247.7
CA 19–9 (U/mL)	2676.9 ± 6783.1
NLR	4.2 ± 3.4
PLR	189.0 ± 95.0

*ECOG* Eastern Cooperative Oncology Group, *BMI* body mass index, *WBC* white blood cell, *CRP* C-reactive protein, *eGFR* estimated glomerluar filtration rate, *AST* aspartate aminotransferase, *ALT* alanine aminotransferase, *ALP* alkaline phosphatase, *CEA* carcinoembryonic antigen, *CA 19–9* carbohydrate antigen 19–9, *NLR* neutrophil-to-lymphocyte ratio, *PLR* platelet-to-lymphocyte ratio

Previous history of curative surgery was identified in 33 (19.1%) patients, extended cholecystectomy in 23 (69.7%), and simple cholecystectomy in 10 (30.3%) patients. Median duration after surgery until recurrence was 10.0 (95% CI 7.79–16.34) months. Median OS in patients with previous history of curative surgery and those without was 7.8 (95% CI 6.7–10.0) and 11.1 (95% CI 6.7–15.1) months, respectively (*P* = 0.202). Twelve patients had previous history of palliative chemotherapy; nine patients received fluoropyrimidine-based chemotherapy, and the other three patients had palliative concurrent chemoradiation therapy with fluoropyrimidine. Among 44 (25.4%) patients who underwent biliary drainage due to malignant hilar obstruction before the initial chemotherapy, 35 (79.5%) were treated with endoscopic retrograde biliary drainage and 9 (20.5%) with percutaneous transhepatic biliary drainage.

### Treatment outcomes

**Fig. 2:**
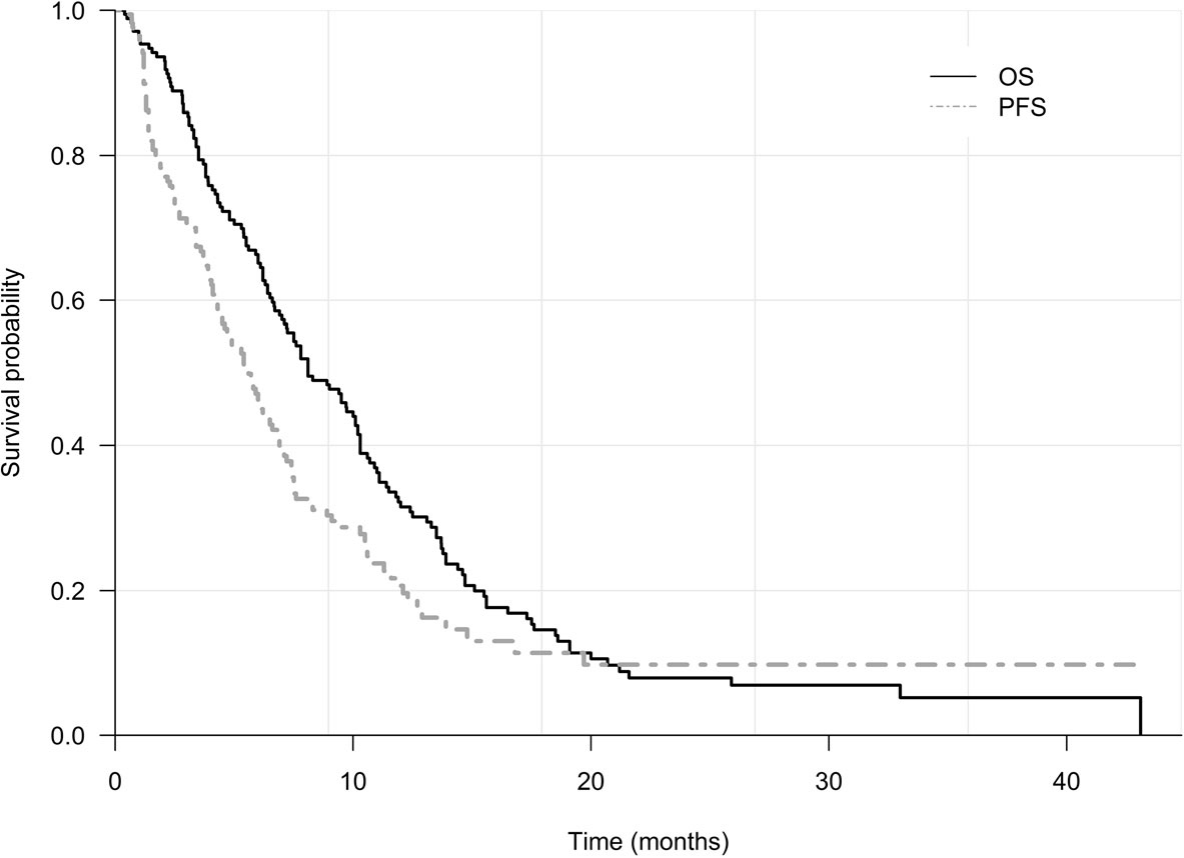
Overall survival (OS) and progression-free survival (PFS) of patients treated with gemcitabine plus cisplatin

Treatment data during GEMCIS chemotherapy are summarised in Table 2. The median follow-up duration was 8.6 ± 7.1 months. Based on 147 (85.0%) deaths, OS was 8.1 (95% CI 7.1–10.2) and PFS was 5.6 (95% CI 4.5–6.8) months (Fig. 2). Disease control was achieved in 103 (59.5%) patients; 2 (1.2%) with complete response (CR), 26 (15.0%) with partial response, and 75 (43.4%) with stable disease. Of the two patients with the best CR response, one patient who initially had metastasis to peritoneum remained in CR until the last date of follow-up and the other patient showed progression of disease with recurrence at the lung 10 months after the last chemotherapy.

**Table 2:** Treatment data and efficacy of GEMCIS in unresectable gallbladder cancer

Variables	*N* = 173
Treatment duration, months	3.8 ± 3.9
Total cycle	5.3 ± 4.4
OS, months (95% CI)	8.1 (7.1–10.2)
PFS, months (95% CI)	5.6 (4.5–6.8)
Best response	
CR	2 (1.2%)
PR	26 (15.0%)
SD	75 (43.4%)
PD	48 (27.7%)
NE	22 (12.7%)
ORR (CR + PR)	28 (16.2%)
DCR (CR + PR + SD)	103 (59.5%)
Number of cycles	
1	38 (22.0%)
2	31 (18.0%)
3	8 (4.7%)
4	16 (9.3%)
5	10 (5.8%)
6	18 (10.5%)
7	6 (3.5%)
8	14 (8.1%)
9	2 (1.2%)
≥ 10	30 (17.4%)

*GEMCIS* gemcitabine plus cisplatin, *OS* overall survival, *PFS* progression-free survival, *CI* confidence interval, *CR* complete response, *PR* partial response, *SD* stable disease, *PD* progressive disease, *NE* not evaluable, *ORR* overall response rate, *DCR* disease control rate

Patients received a total of 5.3 ± 4.4 cycles of chemotherapy during 3.8 ± 3.9 months. A subsequent line of chemotherapy was feasible in 73 (42.2%) patients: fluoropyrimidine-based therapy in 57 (32.9%), clinical trial in 14 (8.1%), gemcitabine single therapy in 2 (1.5%), and concurrent chemoradiation therapy with fluoropyrimidine in 1 (0.7%) patient. Median overall survival of the patients who underwent further lines of palliative chemotherapy was 10.2 (95% CI 8.1–12.0) months, which was not significantly different from that of patients who did not receive a subsequent line of chemotherapy (median OS 6.4 months; 95% CI 4.4–9.7; *P* = 0.086).

With regard to hematological grade 3/4 toxicity, neutropaenia was most commonly noted (28.6%), followed by anaemia (19.3%) and thrombocytopaenia (17.1%). Febrile neutropaenia occurred in eight patients (4.6%). Deep vein thrombosis and pulmonary thromboembolism developed in 18 (10.4%) and 9 (5.2%) patients, respectively. Transaminase increased to more than 3 times the upper limits of normal in 27 (15.6%) patients during treatment. Nausea and/or vomiting (20.2%) and diarrhoea (6.9%) frequently occurred. Severe non-haematological toxicity of grade 3 or greater was infrequent and included diarrhoea (*n* = 3), nausea/vomiting (*n* = 2), and neuropathy (*n* = 1).

### Prognostic factors

In univariable analysis, several variables showed *P* < 0.1 (Table 3): ECOG 2 (HR 1.86; 95% CI 0.94–3.68; *P* = 0.074), invasion to liver (HR 1.47; 95% CI 1.02–2.12; *P* = 0.040), metastasis to liver (HR 1.72; 95% CI 1.23–2.41; *P* = 0.002), metastasis to bone and muscle (HR 2.03; 95% CI 1.14–3.61; *P* = 0.016), total bilirubin≥1.5 X upper limit of normal (HR 1.77; 95% CI 1.21–2.58; *P* = 0.003), transaminase≥1.5 X upper limit of normal (HR 1.53; 95% CI 1.07–2.18; *P* = 0.019), NLR ≥ 3 (HR 2.34; 95% CI 1.66–3.29; *P* < 0.001), PLR ≥ 190 (HR 1.77; 95% CI 1.27–2.47; *P* < 0.001), CEA ≥ 5 ng/mL (HR 1.87; 95% CI 1.33–2.63; *P* < 0.001), and CA 19–9 ≥ 500 U/mL (HR 2.28; 95% CI 1.61–3.23; *P* < 0.001).

**Table 3:** Univariable analysis of possible factors affecting overall survival

	Number of patients (%)	Median OS, months (95% CI)	HR (95% CI)	*P* value
Age				
< 65	90 (52.0%)	10.1 (7.6–11.4)	1.00	
≥ 65	83 (48.0%)	7.2 (6.0–10.0)	1.28 (0.92–1.78)	0.138
Sex (female/ male)				
Female	86 (49.7%)	10.1 (7.8–12)	1.00	
Male	87 (50.3%)	7.1 (5.5–10.1)	1.22 (0.88–1.69)	0.234
ECOG				
0–1	164 (94.8%)	8.1 (7.1–10.3)	1.00	
2	9 (5.2%)	7.5 (2.8-NE)	1.86 (0.94–3.68)	0.074
BMI				
> 25	136 (78.6%)	7.8 (6.6–9.7)	1.00	
≥ 25	37 (21.4%)	11.9 (7.1–15.1)	0.79 (0.53–1.18)	0.255
Stage				
IIIB/ IVA	9 (5.2%)	7.2 (2.1-NE)	1.00	
IVB	164 (94.8%)	8.1 (7.1–10.3)	0.68 (0.35–1.35)	0.272
Charlson comorbidity index				
< 9	110 (63.6%)	9.7 (7.8–11.0)	1.00	
≥ 9	63 (36.4%)	6.4 (4.4–10.2)	1.23 (0.87–1.72)	0.237
Local invasion				
Liver	47 (27.2%)	7.7 (6.6–11.4)	1.47 (1.02–2.12)	0.040
Extrahepatic bile duct	26 (15.0%)	6.5 (5.0–11.9)	1.06 (0.67–1.67)	0.812
Intestine	20 (11.6%)	8.6 (5.0-NE)	0.79 (0.47–1.34)	0.386
Peritoneum	2 (1.2%)	10.5 (2.3-NE)	0.94 (0.23–3.82)	0.933
Hepatic artery	15 (8.7%)	6.4 (5.0-NE)	1.12 (0.63–1.98)	0.699
Portal vein	11 (6.4%)	5.9 (2.3-NE)	1.17 (0.6–2.31)	0.643
Metastasis site				
Liver	74 (42.8%)	6.2 (5.3–10.0)	1.72 (1.23–2.41)	0.002
Peritoneum	46 (26.6%)	6.5 (4.2–10.1)	1.25 (0.87–1.82)	0.229
Lung	29 (16.8%)	7.2 (5.4–15.6)	0.85 (0.54–1.34)	0.487
Bone or muscular system	14 (8.1%)	5.1 (3.3–13.1)	2.03 (1.14–3.61)	0.016
Distant lymph node	102 (59.0%)	8.3 (6.5–10.9)	1.10 (0.79–1.54)	0.560
Total bilirubin				
≤ 1.5 X ULN	130 (75.1%)	9.7 (7.8–11.0)	1.00	
> 1.5 X ULN	43 (24.9%)	5.3 (3.7–9.5)	1.77 (1.21–2.58)	0.003
Transaminase				
≤ 1.5 X ULN	120 (69.4%)	9.7 (7.8–11.5)	1.00	
> 1.5 X ULN	53 (30.6%)	6.2 (4.5–9.7)	1.53 (1.07–2.18)	0.019
NLR				
≤ 3	75 (43.4%)	12.4 (10.2–14.6)	1.00	
> 3	98 (56.6%)	6.2 (4.8–7.8)	2.34 (1.66–3.29)	< 0.001
PLR				
< 190	102 (59.0%)	10.3 (9.4–13.7)	1.00	
≥ 190	71 (41.0%)	6.6 (5.6–8.1)	1.77 (1.27–2.47)	0.001
CEA, ng/mL				
< 5	103 (59.5%)	10.3 (7.8–13.1)	1.00	
≥ 5	70 (40.5%)	6.5 (5.0–8.1)	1.87 (1.33–2.63)	< 0.001
CA 19–9, U/mL				
< 500	114 (65.9%)	10.7 (9.0–13.3)	1.00	
≥ 500	59 (34.1%)	5.5 (4.1–7.6)	2.28 (1.61–3.23)	< 0.001

*OS* overall survival, *HR* hazard ratio, *ECOG* Eastern Cooperative Oncology Group, *BMI* body mass index, *NLR* neutrophil-to-lymphocyte ratio, *PLR* platelet-to-lymphocyte ratio, *CEA* carcinoembryonic antigen, *CA 19–9* carbohydrate antigen 19–9, *NE* not estimable, *ULN* upper limit of normal

**Fig. 3:**
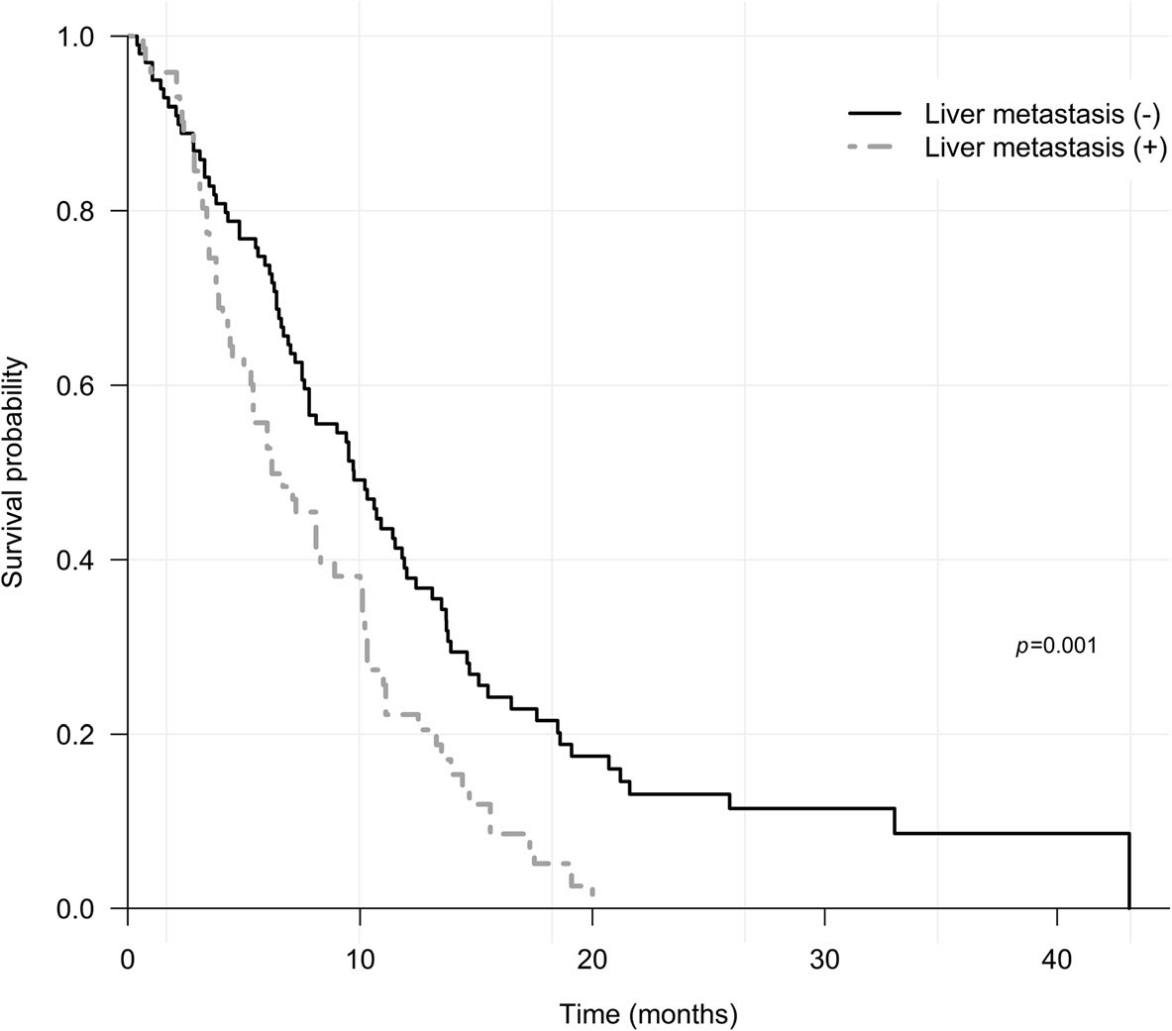
Kaplan–Meier curves of overall survival according to liver metastasis

**Fig. 4:**
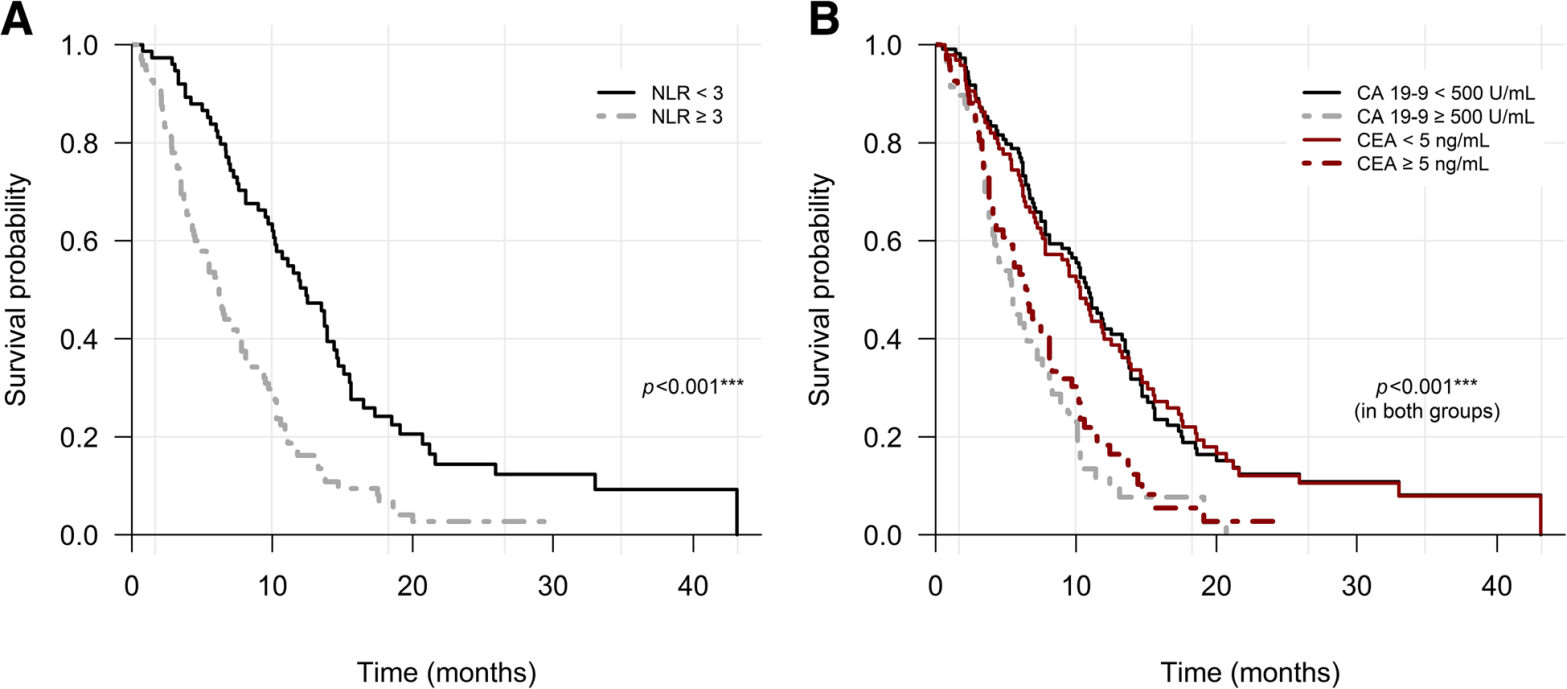
Kaplan–Meier curves of overall survival according to groups based on the results of multivariable analysis and log-rank test. **a** Classified by NLR ≥3. **b** Classified by CA19–9 ≥ 500 U/mL and CEA ≥ 5 ng/mL. NLR, neutrophil-to-lymphocyte ratio; CA 19–9, carbohydrate antigen 19–9; CEA, carcinoembryonic antigen

The results of multivariable analysis are listed in Table 4. In the final multivariable analysis, independent prognostic factors for poor OS were metastasis to liver (HR 1.63; 95% CI 1.11–2.40; *P* = 0.013), NLR ≥ 3 (HR 1.65; 95% CI 1.09–2.49; *P* = 0.017), CEA ≥ 5 ng/mL (HR 1.50; 95% CI 1.02–2.19; *P* = 0.038) and CA19–9 ≥ 500 (HR 1.59; 95% CI 1.01–2.50; *P* = 0.043). Median OS in patients with liver metastasis was 6.2 (95% CI 5.3–10.0, *P* = 0.001) months (Fig. 3). Hypertransaminasemia was identified in 26/74 (35.1%) patients in patients with liver metastasis, which was not significantly different from the incidence in patients without liver metastasis (*P* = 0.346). When classified by baseline NLR ≥ 3, CA 19–9 ≥ 500 U/mL and CEA ≥ 5 ng/mL, median OS was 6.2 (95% CI 4.8–7.8, *P* < 0.001), 5.5 (95% CI 4.1–7.6, *P* < 0.001) and 6.5 (95% CI 5.0–8.1, *P* < 0.001) months, respectively (Fig. 4).

**Table 4:** Multivariable analysis by Cox regression model

Variable	Number	HR (95% CI)	*P*-value
ECOG			
0–1	164	1.00	
2	9	0.79 (0.34–1.85)	0.586
Invasion to liver			
No	126	1.00	
Yes	47	0.91 (0.59–1.39)	0.648
Metastasis to liver			
No	99	1.00	
Yes	74	1.63 (1.11–2.40)	0.013
Metastasis to bone and muscle			
No	159	1.00	
Yes	14	1.78 (0.93–3.42)	0.081
NLR			
< 3	75	1.00	
≥ 3	98	1.65 (1.09–2.49)	0.017
PLR			
< 190	102	1.00	
≥ 190	71	1.19 (0.79–1.77)	0.405
Total bilirubin			
≤ 1.5 X ULN	130	1.00	
> 1.5 X ULN	43	1.56 (0.95–2.55)	0.077
Transaminase			
≤ 1.5 X ULN	120	1.00	
> 1.5 X ULN	53	0.92 (0.59–1.45)	0.723
CEA, ng/mL			
< 5	103	1.00	
≥ 5	70	1.50 (1.02–2.19)	0.038
CA 19–9, U/mL			
< 500	114	1.00	
≥ 500	59	1.59 (1.01–2.50)	0.043

*ECOG* Eastern Cooperative Oncology Group, *CEA* carcinoembryonic antigen, *CA 19–9* carbohydrate antigen 19–9, *NLR* neutrophil-to-lymphocyte ratio, *PLR* platelet-to-lymphocyte ratio, *CEA* carcinoembryonic antigen, *CA 19–9* carbohydrate antigen 19–9, *ULN* upper limit of normal

## Discussion

The aim of this study was to examine the efficacy of GEMCIS and identify prognostic factors in unresectable GBC. In this study, GEMCIS showed a high disease control rate, and liver metastasis, NLR, CEA, and CA 19–9 were significantly associated with prognosis. Overall, this study showed a median OS of 8.6 months, in general accordance with the previous phase II study in Japan regarding the efficacy of GEMCIS in 14 patients with unresectable GBC [4]. On the other hand, previous study which investigated efficacy of GEMCIS in patients with BTC reported the median OS of 11.7 months, suggesting that prognosis is worse in GBC than that in other subtypes of BTC [4, 5]. Moreover, in this study, there were 76 patients who were not able to undergo chemotherapy, 48 patients loss to follow-up, and 4 patients who died immediately after GBC diagnosis. Since GEMCIS was clinically applied in patients with generally good performance, the actual prognosis of unresectable GBC is likely to be worse than the prognosis reported in this study.

The gallbladder has no serosal layer near the liver and its perimuscular connective tissue is in direct contact with the liver. Subsequently, invasion of GBC into liver is very common and liver involvement is known to be associated with poor prognosis [3, 6]. In this study, however, liver invasion was not an independent prognostic factor, as opposed to previous studies which mostly included patients with resectable GBC. The discrepancy may stem from the different inclusion criteria. Meanwhile, metastasis to liver, which occurs via portal tracts, is common in advanced GBC and indicates poor prognosis [9, 20, 21]. These findings coincide well with the results of our study that identified liver metastasis as an independent poor prognostic factor. Currently, there are no standard treatment methods regarding liver metastasis although several chemotherapy regimens can be considered in patients with liver metastasis on the basis of the National Comprehensive Cancer Network Clinical Practice Guidelines [22]. Because of the absence of the specific standard treatment guidelines, further studies are required to evaluate best treatment modalities for liver metastasis.

It is well known that CEA and CA19–9 are helpful not only in diagnosis, but also in predicting prognosis of GBC [11, 12]. Wang et al. [13] reported that CA19–9 plays an important role as an independent prognostic factor in GBC. Likewise, Park et al. [23] recently reported that CEA independently predicts prognosis in patients with metastatic BTC. Consistent with previous studies [23, 24], the results of our study demonstrated that baseline CA 19–9 and CEA level were independent prognostic markers.

The association of NLR with prognosis has been widely studied in patients with BTC [15, 16, 23, 25]. Zhang et al. [14] found that patients with NLR ≥ 2.61 had a worse prognosis than those with NLR < 2.61 in a study of 316 patients with GBC treated with surgery. In our study, multivariate analysis revealed NLR ≥ 3 as an independent risk factor for poor OS, which was in general accordance with previous studies. The correlation between NLR and prognosis can be explained by the fact that neutrophils secrete vascular endothelial growth factors and several cytokines to promote tumour development and proliferation whereas lymphocytes play a crucial role in tumour defence by inducing cytotoxic cell death [26–28]. Pro-inflammatory and pro-angiogenic cytokines are known to be important causative factors in the development of BTC and cytokine-based therapies have been studied [29]. It is expected that the NLR will play an important role in the prediction of prognosis in future development of therapies targeting cancer-associated inflammation.

A limitation of our study is that it is based on retrospective data from a single tertiary care center. Although the results of our study showed a somewhat longer OS than the previous phase II clinical trial in Japan, there may be several confounding factors stemming from the retrospective study design that affect the results of analysis. Second, since the present study was conducted without a control group, careful interpretation and further validation is needed. Despite these limitations, clinical data from this study are helpful because there are very few studies examining the efficacy of GEMCIS combination chemotherapy and prognostic factors specifically in patients with unresectable GBC.

Third, the cut-off values of the tumour markers, NLR, and PLR were estimated based on a relatively small sample size. Although these cut-off values were found to be statistically significant, a large-scale prospective study to determine the ideal cut-off value is needed. Another limitation is that direct invasion of GBC to surrounding tissues was based on imaging findings because we selected only patients with unresectable disease. Diagnostic aspects of imaging tests in evaluating direct invasion of tumour to surrounding tissues may be less sensitive or less accurate than pathologic examination accompanied by surgical resection. Nevertheless, due to the development of recent imaging techniques, the sensitivity and specificity of the diagnosis by computed tomography are as high as 99 and 76%, respectively, and the interpretation of the results of this study seems to be reasonable [30].

## Conclusions

In conclusion, GEMCIS is an effective regimen in patients with unresectable GBC. The prognostic factors identified in this study might help accurate patient risk stratification and decision of a proper treatment plan.

## Abbreviations

BTC: Biliary tract cancer
CA19–9: Carbohydrate antigen 19–9
CEA: Carcinoembryonic antigen
CI: Confidence interval
ECOG: Eastern Cooperative Oncology Group
GBC: Gallbladder cancer
GEMCIS: Gemcitabine plus cisplatin
HR: Hazard ratio
IQR: Interquartile range
NLR: Neutrophil-to-lymphocyte ratio
OS: Overall survival
PFS: Progression-free survival
PLR: Platelet-to-lymphocyte ratio
